# Prevalence of gastrointestinal parasites in three groups of domestic poultry managed under backyard system in the Savanna subregion, Department of Sucre, Colombia

**DOI:** 10.5455/javar.2021.h551

**Published:** 2021-11-01

**Authors:** Donicer Eduardo Montes-Vergara, José Cardona-Alvarez, Alexander Pérez-Cordero

**Affiliations:** 1 Faculty of Agricultural Sciences, University of Sucre, Sincelejo, Colombia; 2 Department of Veterinary Medicine and Zootechnics, University of Córdoba, Monteria, Colombia

**Keywords:** Gastrointestinal parasites, prevalence, backyard poultry, *Ascaridia*, *Heterakis*, *Capillaria*, *cestodes*

## Abstract

**Objective:**

To identify the prevalence of gastrointestinal parasites that affect the backyard poultry system in the Savanna region, Department of Sucre, Colombia.

**Materials and Methods:**

Fecal samples were collected from 860 native birds, both hens (*Gallus domesticus*), ducks (*Anas platyrhynchos domesticus*), and turkeys (*Meleagris gallopavo*), regardless of age and sex. Samples were processed using direct techniques with ZnSO_4_ and indirect methods such as modified Sloss. Data were presented as frequencies, and the nonparametric odds ratio test was used for two independent samples.

**Results:**

A total of 77.3% (665/860) of the birds were infected with one or more species of gastrointestinal parasites. Among the nematodes, *Capillaria* spp. (45.6%), *Ascaridia galli* (18.4%), *Heterakis gallinarum* (59.4%), *Syngamus trachea* (38.9%), *Tetrameres* spp. (25.2%), and *Strongylus* spp. (12.2%) were recorded. The cestodes were *Choanotaenia infundibulum* (22.6%), *Davainea proglottina* (42.3%), *Raillietina* spp. (58.3%), and *Hymenolepis* spp. (54.7%), while only *Eimeria* spp. (90%) was recorded as protozoa.

**Conclusions:**

The study showed a high incidence of gastrointestinal parasite infestations, the most common species being *Hymenolepis* spp., *Eimeria* spp., *Raillietina* spp., and *Heterakis gallinarum*.

## Introduction

Backyard or rustic “family poultry farming” is a form of traditional domestic breeding that requires few inputs and includes a variety of bird species such as chickens, turkeys, ducks, geese, and quails [1], and is the most traditional and widespread livestock activity in rural communities, as it benefits rural families by providing high-nutrient products such as meat and eggs, as well as revenue from surpluses [2]. The poultry population in Colombia is distributed among 440,381 farms, of which 98.7% are backyard farms, and the remaining 1.3% correspond to technological farms. Of the total number of birds in Colombia (210,541,160), 95.8% are technified poultry and 4.2% are backyard poultry. The backyard poultry population in 2021 was more than 8 million, of which 856,764 were registered in the department of Sucre, Colombia [3].

Backyard poultry farming is an important economic activity for the rural population as a source of income and as a way to guarantee food security in unprotected communities [4]. In this sense, support for backyard poultry farming has been widely used since it is considered that small-scale livestock production represents an effective alternative to achieving food security [5]. Farm animals constitute an essential element of subsistence for the rural poor, performing multiple functions: food production, fertilizer, and income generation [6].

Birds managed in BPS are vulnerable to parasite attacks [7]. The occurrence of parasitic infestations has a high prevalence, causing low economic conditions, increased mortality and prophylaxis, leading to low production, death of animals, and limited productivity. Improved poultry management practices are responsible for the reduction in the incidence of parasitic infections [8]. Thus, the objective of this work was to identify the prevalence of gastrointestinal parasites in *Gallus domesticus*, *Anas platyrhynchos domesticus*, and *Meleagris gallopavo* managed under the backyard poultry system in the Savanna subregion, Department of Sucre, Colombia.

## Materials and Methods

**Figure 1.**
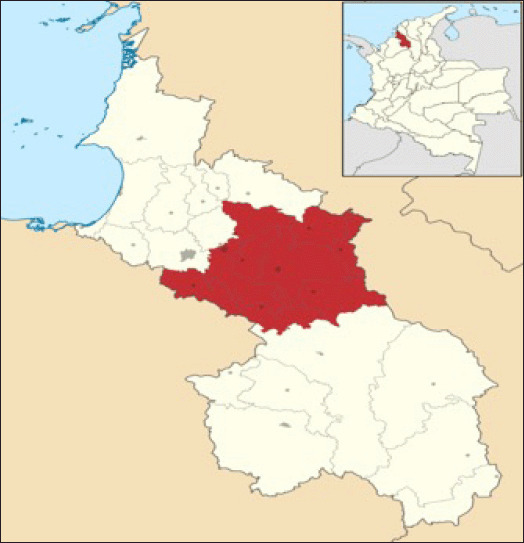
Location of the study area. Savanna sub-region, Department of Sucre Colombia. Wikipedia, the free encyclopedia. Accessed on: November 1, 2021, from https://es.wikipedia.org/w/index.php?title=Subregi%C3%B3n_de_La_Sabana_(Sucre)&oldid=135872197.

The study was conducted in the Savanna subregion (Sincelejo, Sincé, El Roble, San Pedro, Sampués, Los Palmitos, Galeras, Buenavista, Corozal and San Juan de Betulia) of the Department of Sucre, Colombia (Fig. 1) between January 2019 and March 2020. The climate of the study area is characteristic of tropical dry forest (bst) zones, with an average annual temperature of 27.2°C, yearly rainfall between 990 and 1,275 mm, and relative humidity of 80% [9]. The region is characterized by commercial poultry farms and numerous backyard poultry operations.

The Savannas subregion in the Department of Sucre, Colombia, includes 10 municipalities and has a backyard poultry census of 42,624, distributed among 794 farms [3]. The sample size was determined according to Suarez and Tapia [10], using a confidence level of 95% (*Z* = 1.96) and a maximum margin of error of 10%, resulting in a total of 85.6 farms. Thus, an average of 8.6 farms per municipality was visited, using stratified random sampling, in which 860 adult criollo birds were characterized, considering environmental, sanitary, and management factors. 

We worked with four geographical groups, which were characterized by their relative proximity (Group 1 = Sincelejo, Los Palmitos, Corozal; Group 2 = Sincé, Galeras, S-Betulia; Group 3 = El Roble, Sampués; Group 4 = San Pedro, Buenavista), in order to establish prevalence levels by geographical group.

A cross-sectional coprological study of feces from hens (*G. domesticus*), ducks (*A. platyrhynchos domesticus*), and turkeys (*M. gallopavos*) was carried out. The samples were taken in the morning hour before feeding. The animals evaluated on each farm were placed in a portable cage according to the group under study. Black plastic was placed on the floor at the bottom of the cage to avoid feces contamination with the ground. Once the birds had deposited their feces, a swab was taken, and the sample was collected in a sterile fecal matter collection container. The sample was kept cold at 5°C for subsequent analysis in the Microbiology I laboratory of the University of Sucre.

A total of 860 fecal samples were processed and analyzed using direct techniques with ZnSO4 and indirect methods such as modified Sloss [11], quantified with MacMaster chamber [8,12]. To calculate the frequency of gastrointestinal parasite infection, descriptive statistics using absolute and relative frequency were used. The nonparametric *odds ratio* test for two independent samples was used to determine the risk factors with a significance level of 5% in each geographical group. The variables were subjected to the Shapiro–Wilk test as a normality distribution test (*p* < 0.05). The presence of parasites by the geographic group was compared using the Kruskal–Wallis test for nonparametric variables and Dunn’s test to determine differences between groups [13]. Statistical analysis was carried out with the R program.

## Results and Discussion

A total of 77.3% (665/860) of the samples were positive for gastrointestinal parasites, with one protozoan species, four cestodes species, and six nematodes species. The latter were *Capillaria* ssp. (45.6%), *Ascaridia galli* (18.4%), *Heterakis gallinarum* (59.4%), *Syngamus* *trachea* (38.9%), *Tetrameres* spp. (25.2%), and *Strongylus* (12.2%). The cestodes corresponded to *Choanotaenia* *infundibulum* (22.6%), *Davainea* *proglottina* (42.3%), *Raillietina* spp. (58.3%), and *Hymenolepis* spp. (54.7%). The protozoan recorded was *Eimeria* spp. (90%) (Table 1).

The high prevalence rate could be due to the fact that birds are kept in absolute freedom, feeding on many agricultural byproducts, pastures, and intermediate hosts of parasites (beetle, grasshoppers, cockroaches, crustaceans, earthworm, and snail) available in the environment [11,14].

The prevalence of gastrointestinal parasite infections varies between regions and countries [15]. For example, prevalences between 97.6% and 99.2% were reported in Germany [16], 99.3% in Italy [17], 72% in Iran [18,19], 73.9% in Thailand [20], and 95.8% in Zambia [21], with environmental conditions, age of birds, and availability of intermediate hosts being the main determinants for the variability of parasite prevalence [22].

**Table 1.** Frequency of gastrointestinal parasites in domestic poultry (*n* = 860) managed under the backyard system in the Savanna subregion, Department of Sucre, Colombia.

GP	Species	Frequency (%)	IC 95%
Protozoa	*Eimeria* spp.	90.0	82.5–94.4
Cestodes	*C. infundibulum*	22.6	15.5–31.7
	*D. proglottina *	42.3	33.0–52.0
	*Raillietina* spp.	58.3	48.5–67.4
	*Hymenolepis* spp.	54.7	44.9–64.1
Nematodes	*Capillaria* ssp.	45.6	36.1–55.3
	*A. galli*	18.4	12.0–27.1
	*H. gallinarum*	59.4	49.6–68.5
	*S. trachea*	38.9	29.9–48.6
	*Tetrameres* spp.	25.2	17.7–34.5
	*Strongylus *spp.	12.2	7.14–20.0

GP = parasitic group.

The most prevalent parasite in the three groups was *Eimeria* spp, with a prevalences of 87.4%, 91.2%, and 90.2% in hens, ducks, and turkeys, respectively. The frequency of parasites according to avian species is shown in Table 2. The high prevalence of *Eimeria* spp. is a product of poor sanitation and poor management of the poultry habitat. Studies in Nigeria report the presence of *Eimeria* spp. as the most prevalent protozoan among gastrointestinal parasites infecting poultry [23–25].

*Raillietina* spp. was the most prevalent among the cestodes in the studied population; however, it was not observed in turkeys. *Hymenolepis* spp. predominated (Table 2) the most common type of parasite infecting domestic fowl [26–29].

The prevalence of nematodes ranged from 10.2% to 68.6% in the study birds (Table 2). Reports of nematode prevalence in domestic poultry managed under the SAT scheme have been reported [30–33] in various countries. Among the nematodes encountered, *H. gallinarum* is not pathogenic. Still, it is a vector of *Histomonas meleagridis*, which is highly pathogenic and involved in “*black head*” disease, which is lethal to many domestic fowl [34].

Nematode parasitism changes the gastrointestinal system of chickens, resulting in decreased performance and, in some cases, mortality [35,33]. While there is no information on the direct economic costs of nematode infestation in the Savanna, Sucre sub-region, the prevalence discovered warrants the use of mitigation techniques to offset the detrimental impact on SAT productivity. 

**Table 2.** Frequency of gastrointestinal parasites in domestic poultry managed in the backyard systems in the Savanna subregion, Department of Sucre, Colombia.

Poultry	Samples		Parasites		Frequency (%)
	*n*	Positive	Groups	Species	
Hens	535	414	Protozoa	*Eimeria* spp.	87.4
			Cestodes	*Raillietina* spp.	45.5
				*Hymenolepis* spp.	28.3
				*D. * *proglottina*	55.1
			Nematodes	*Capillaria* spp.	31.2
				*A. galli*	26.8
				*H. gallinarum*	39.6
				*S. trachea*	24.2
Ducks	179	138	Protozoa	*Eimeria *spp.	91.2
			Cestodes	*Raillietina* spp.	64.6
				*Hymenolepis* spp.	26,7
			Nematodes	*Capillaria* ssp.	22.3
				*A. galli*	36.2
				*H. gallinarum*	28.4
				*S. trachea*	38.2
Turkeys	146	113	Protozoa	*Eimeria* spp.	90.2
			Cestodes	*C. infundibulum*	22.6
				*Hymenolepis* spp.	76.1
			Nematodes	*Capillaria* ssp.	68.2
				*A. galli*	10.2
				*H. gallinarum*	68.6
				*S. trachea*	45.9
				*Tetrameres* spp.	25.2
				*Strongylus *spp.	12.3
Total	860	665			

The high prevalence rate in the study area may be a result of poor sanitary conditions, high poultry population density, uncontrolled feeding, and a lack of attention to treatment and disease control and prevention measures, all of which expose birds to poor hygiene on farms and in poultry houses, allowing them to contract a wide variety of harmful parasites [36].

Chemical control of parasites is simple, inexpensive, and can be used both therapeutically and prophylactically. However, chemical treatment has several drawbacks, such as weakening natural immunity and the presence of residues in food and the environment. In addition, chemical anthelmintics can stimulate resistance, so alternative forms of control are needed [8].

**Table 3.** Distribution and prevalence of gastrointestinal parasites according to geographic location in the Savanna subregion, Department of Sucre, Colombia.

Grups	Municipality	Total	Positive		OR	IC -95%	*p*-value
			%	%			
1	Sincelejo	85	72.9	68.2	0.87	0.66–1.14	0.34
	Los Palmitos	90	64.4				
	Corozal	92	67.4				
2	Sincé	90	72.2	78.1			
	Galeras	83	88.0				
	SJ de Betulia	73	74.0				
3	El Roble	82	84.1	82.1	0.97	0.71–1.33	0.93
	Sampués	87	81.6				
4	San Pedro	94	84.0	84.8			
	Buenavista	84	85.7				
Total		860	77.3				

The prevalence of gastrointestinal parasites was high in all the study municipalities, regardless of geographic location (Table 3), without significant differences. Education and motivation of farmer producers on biosecurity measures may aid in mitigating the negative effects of parasitic infection on poultry response effectiveness [33,37].

## Conclusion

Gastrointestinal parasites are endemic among the domestic poultry managed under the backyard system in the Savanna region, Sucre, Colombia, showing a high prevalence of gastrointestinal parasites, the most frequent being *Hymenolepis* spp., *Eimeria* spp., *Raillietina *spp., and *H. gallinarum*, in the period studied. It is essential to know the conditions of the farm to develop the best prevention program, allowing the recognition of the factors that influence the possibility of disease incidence.

## List of abbreviations

BPS, backyard poultry system; GP, parasitic group; IC, confidence interval; n, sample size; OR, Odds ratio; p, probability value; spp., species.
